# Níveis Séricos de Glicogênio Sintase 3 Beta: Um Marcador Promissor para Pacientes com Insuficiência Cardíaca

**DOI:** 10.36660/abc.20240684

**Published:** 2024-11-19

**Authors:** Claudia Aznar Alesso, João Queda, Dania Mohty, Vera Maria Cury Salemi

**Affiliations:** 1 Centro Universitário Fieo UNIFIEO São Paulo SP Brasil Centro Universitário Fieo - UNIFIEO, São Paulo, SP – Brasil; 2 Faculdade de Medicina de Santo Amaro São Paulo SP Brasil Faculdade de Medicina de Santo Amaro, São Paulo, SP – Brasil; 3 Hospital Universitário Dupuytren Departamento de Cardiologia Limoges França Departamento de Cardiologia, Hospital Universitário Dupuytren, Limoges – França; 4 Hospital Especializado e Centro de Pesquisa King Faisal Centro do Coração Riad Arábia Saudita Centro do Coração, Hospital Especializado e Centro de Pesquisa King Faisal, Riad – Arábia Saudita; 5 Hospital Sírio Libanês São Paulo SP Brasil Hospital Sírio Libanês, São Paulo, SP – Brasil

**Keywords:** Glicogênio, Biomarcadores, Insuficiência Cardíaca, Diagnóstico, Prognóstico

A insuficiência cardíaca (IC) envolve inflamação sistêmica e alterações metabólicas. A prevalência está aumentando devido a melhorias nos tratamentos de IC e maior expectativa de vida na população.^1^ Além disso, a abordagem diagnóstica de pacientes com IC aumentou significativamente com o desenvolvimento de novos algoritmos para promover o diagnóstico mais precoce.^1^

O uso cuidadoso de biomarcadores pode ajudar a refinar o diagnóstico e o tratamento e melhorar ainda mais o prognóstico de pacientes com IC. O termo "biomarcador" (de marcador biológico) foi cunhado em 1989 para identificar um parâmetro biológico mensurável e quantificável usado para avaliar a saúde e a fisiologia dos pacientes em termos de risco de doença e diagnóstico.^2^ Desde os primeiros estudos de Braunwald na década de 1950 sobre a proteína C-reativa (PCR) na IC, centenas de moléculas foram estudadas, mas apenas o peptídeo natriurético tipo B (BNP) e o pró-peptídeo natriurético tipo B N-terminal se aproximam das características do biomarcador ideal da IC.^2^ No entanto, confiar em biomarcadores de congestão facilmente mensuráveis, como BNPs, deixa de detectar aproximadamente um terço de todos os pacientes afetados, podendo ser desproporcionalmente alto para pacientes com insuficiência renal, hipertensão pulmonar, embolia pulmonar e hipóxia crônica.^3-5^

Na última década, o otimismo tem sido alto quanto ao potencial significado clínico da glicogênio sintase quinase-3 (GSK-3) como um alvo farmacológico para isquemia e IC.^6^ É uma serina-treonina quinase, que foi identificada pela primeira vez como um regulador do metabolismo do glicogênio e da homeostase da glicose. Além disso, está envolvida em uma variedade de processos biológicos para manter a homeostase cardíaca normal, incluindo proliferação celular, apoptose, sinalização de insulina, fibrose, hipertrofia, controle da divisão celular e desenvolvimento cardíaco da resposta imune. Em pacientes com IC, sua inibição induz inibição da apoptose e pode apresentar um mecanismo protetor.^7-10^ GSK3 é codificada por dois genes diferentes, alfa (α) e beta (β). Além disso, esses genes codificam um conjunto de uma variante de splicing alfa (α) e duas beta (β1 e β2).

Vários mecanismos podem regular a GSK3, e sua atividade basal está sujeita à ativação ou inibição.^11^ Em resposta a um infarto do miocárdio, um objetivo imediato é restringir a expansão da cicatriz e preservar a função. Além disso, fortes evidências agora apoiam um papel para a inibição da GSK-3 mediando o poro de transição da permeabilidade mitocondrial no mecanismo de proteção do pré-condicionamento isquêmico. No coração, a ênfase foi colocada particularmente na GSK3β em vez da GSK3α, pois a GSK-3β é regulada negativamente na hipertrofia e na IC e atua como um regulador negativo.^9,12^

O elegante estudo prospectivo publicado no ABC-2024-0155 da presente edição mostra que 112 pacientes com IC crônica apresentam níveis aumentados de GSK3β em comparação com 50 controles saudáveis. À medida que a fração de ejeção do ventrículo esquerdo reduz, os níveis de GSK3β também diminuem para prevenir a apoptose e a morte dos miócitos, mostrando um mecanismo protetor.^13^ Os autores sugerem que ele poderia ser usado como um marcador de IC em associação com BNP. No entanto, o BNP apresenta um aumento importante na IC com fração de ejeção reduzida, enquanto a GSK3β aumenta em pacientes com IC com fração de ejeção preservada. A avaliação da diástole neste estudo não incluiu o volume do átrio esquerdo, que apresenta um papel importante em pacientes com IC. Por outro lado, marcadores de inflamação como PCR, razão neutrófilo-linfócito e razão plaquetas/linfócitos não apresentaram diferenças estatísticas em comparação ao grupo controle. Concluímos que mais estudos são necessários para mostrar o valor da GSK3β em pacientes com IC e a associação de biomarcadores na prática clínica.
