# Supplementary material for: Níveis Séricos de Glicogênio Sintase 3 Beta: Um Marcador Promissor para Pacientes com Insuficiência Cardíaca
Source: Arq Bras Cardiol. 2024 Nov 19;121(11):e20240684. [Article in Portuguese] doi: 10.36660/abc.20240684 (PMC11634300; doi:10.36660/abc.20240684)
Supplement: Translation

# Translation: Serum Glycogen Synthase 3 Beta Levels: A Promissory Marker for Patients with Heart Failure

**DOI:** 10.36660/abc.20240684i

**Authors:** Claudia Aznar Alesso, João Queda, Dania Mohty, Vera Maria Cury Salemi

**Affiliations:** 1 São Paulo SP Brazil Centro Universitário Fieo - UNIFIEO, São Paulo, SP – Brazil; 2 São Paulo SP Brazil Faculdade de Medicina de Santo Amaro, São Paulo, SP – Brazil; 3 Limoges France Departamento de Cardiologia, Hospital Universitário Dupuytren, Limoges – France; 4 Riad Saudi Arabia Centro do Coração, Hospital Especializado e Centro de Pesquisa King Faisal, Riad – Saudi Arabia; 5 São Paulo SP Brazil Hospital Sírio Libanês, São Paulo, SP – Brazil

**Keywords:** Glycogen, Biomarkers, Heart Failure, Diagnosis, Prognosis

Heart failure (HF) involves systemic inflammation and metabolic alterations. The prevalence is rising due to improvements in HF treatments and longer life expectancy in the population.^1^ Also, the diagnostic approach of patients with HF has markedly increased with the development of new algorithms to promote earlier diagnosis.^1^

Careful use of biomarkers might help to refine the diagnosis and management and further improve the prognosis of HF patients. The term "biomarker" (from biological marker) was coined in 1989 to identify a measurable and quantifiable biological parameter used to assess the health and physiology of patients in terms of disease risk and diagnosis.^2^ Since Braunwald's first studies in the 1950s on C-reactive protein (CRP) in HF, hundreds of molecules have been studied, but only B-type natriuretic peptide (BNP) and N-terminal pro–B-type natriuretic peptide come close to the characteristics of ideal HF biomarker.^2^ However, relying on easily measurable congestion biomarkers such as BNPs misses approximately one-third of all affected patients. It may be disproportionately high for patients with renal failure, pulmonary hypertension, pulmonary embolism, and chronic hypoxia.^3-5^

Over the past decade, optimism has been high for the potential clinical significance of glycogen synthase kinase-3 (GSK-3) as a pharmacological target for ischemia and HF.^6^ It is a serine-threonine kinase, which was first identified as a regulator of glycogen metabolism and glucose homeostasis. Also, it is involved in a variety of biological processes to maintain normal cardiac homeostasis, including cell proliferation, apoptosis, insulin signaling, fibrosis, hypertrophy, the control of cell division, and immune response cardiac development. In HF patients, its inhibition induces apoptosis inhibition and may present a protective mechanism.^7-10^ GSK3 is encoded by two different genes, alpha (α) and beta (β). Moreover, these genes encode a set of one alpha (α) and two beta (β1 and β2) splice variants.

Multiple mechanisms may regulate GSK3, and its basal activity is subjected to activation or inhibition.^11^ In response to a myocardial infarction, an immediate goal is to restrict scar expansion and preserve function. Also, strong evidence now supports a role for GSK-3 inhibition mediating the mitochondrial permeability transition pore in the mechanism of protection of ischemic preconditioning. In the heart, emphasis has been placed particularly on GSK3β rather than GSK3α, as GSK-3β is downregulated in hypertrophy and HF and acts as a negative regulator.^9,12^

The elegant prospective study published in ABC-2024-0155 of the present issue shows that 112 patients with chronic HF present increased levels of GSK3β compared to 50 healthy controls. As the left ventricle ejection fraction reduces, the levels of GSK3β also decrease to prevent apoptosis and myocyte death, showing a protective mechanism.^13^ The authors suggest that it could be used as a marker of HF in association with BNP. However, BNP presents an important increase in HF with reduced ejection fraction, whereas GSK3β rises in patients with HF with preserved ejection fraction. The evaluation of diastole in this study did not include left atrium volume, which presents an important role in HF patients. On the other side, markers of inflammation such as CRP, neutrophil-lymphocyte ratio, and platelets to lymphocyte ratio did not present statistical differences compared to the control group. We conclude that further studies are needed to show the value of GSK3β in HF patients and the association of biomarkers in clinical practice.
